# Anlotinib Inhibits Cisplatin Resistance in Non-Small-Cell Lung Cancer Cells by Inhibiting MCL-1 Expression via MET/STAT3/Akt Pathway

**DOI:** 10.1155/2024/2632014

**Published:** 2024-03-04

**Authors:** Lile Wang, Lu Xu, Shuhua Han, Xiaoli Zhu

**Affiliations:** ^1^Department of Respiratory Medicine, Zhongda Hospital, School of Medicine, Southeast University, Nanjing 210009, China; ^2^School of Medicine, Southeast University, Nanjing 210009, China

## Abstract

**Background:**

Anlotinib is an effective targeted therapy for advanced non-small-cell lung cancer (NSCLC) and has been found to mediate chemoresistance in many cancers. However, the underlying molecular mechanism of anlotinib mediates cisplatin (DDP) resistance in NSCLC remains unclear.

**Methods:**

Cell viability was assessed by the cell counting kit 8 assay. Cell proliferation, migration, and invasion were determined using the colony formation assay and transwell assay. The mRNA expression levels of mesenchymal-epithelial transition factor (MET) and myeloid cell leukemia-1 (MCL-1) were measured by quantitative real-time PCR. Protein expression levels of MET, MCL-1, and STAT3/Akt pathway-related markers were examined using western blot analysis.

**Results:**

Our data showed that anlotinib inhibited the DDP resistance of NSCLC cells by regulating cell proliferation and metastasis. Moreover, MET and MCL-1 expression could be decreased by anlotinib treatment. Silencing of MET suppressed the activity of the STAT3/Akt pathway and MCL-1 expression. Furthermore, MET overexpression reversed the inhibitory effect of anlotinib on the DDP resistance of NSCLC cells, and this effect could be eliminated by MCL-1 knockdown or ACT001 (an inhibitor for STAT3/Akt pathway).

**Conclusion:**

Our results confirmed that anlotinib inhibited DDP resistance in NSCLC cells, which might decrease MCL-1 expression via mediating the MET/STAT3/Akt pathway.

## 1. Introduction

Non-small-cell lung cancer (NSCLC) is the most common type of lung cancer, accounting for 80% of the total lung cancer [1, 2]. NSCLC is characterized by easy recurrence, micrometastasis, and poor prognosis, so its treatment is very difficult [3, 4]. At present, chemotherapy and molecular targeted therapy are the main treatment methods for NSCLC, but the occurrence of drug resistance is a major problem to be solved in clinical practice [5–7]. Therefore, it is of great significance to explore the molecular mechanism of drug resistance for effective treatment of NSCLC.

Anlotinib, a novel multitarget tyrosine kinase inhibitor (TKI), has inhibition effects on tumor angiogenesis and growth [8, 9]. In addition to being used as a third-line treatment for patients with advanced NSCLC [10], the antitumor effects of anlotinib have been found in many other types of cancer, such as oral squamous cell carcinoma [11] and thyroid cancer [12]. Mesenchymal-epithelial transition factor (MET) is a receptor tyrosine kinase that regulates cell proliferation, apoptosis, metastasis, and drug resistance [13]. Some research studies have shown that MET is overexpressed in osteosarcoma tumor tissues, and anlotinib suppresses osteosarcoma progression and cisplatin (DDP) resistance by reducing MET expression and phosphorylation [14, 15]. Moreover, anlotinib has been confirmed to decrease MET expression and phosphorylation to hinder colorectal carcinoma cell growth and oxaliplatin resistance [16]. A recent study suggests that anlotinib combined with osimertinib therapy represses the osimertinib resistance in NSCLC [17]. However, whether anlotinib mediates NSCLC progression and DDP resistance through MET-related pathways has not been studied.

It has been reported that silencing of CSE1L inhibits lung cancer proliferation and metastasis through the MET/STAT3/PD-L1 pathway [18]. Besides, MET knockdown suppresses pancreatic ductal adenocarcinoma proliferation and metastasis by inhibiting the Akt-related signaling activity [19]. In osteosarcoma, MET has been shown to regulate osteosarcoma progression by mediating the activity of the STAT3/Akt pathway [14]. Therefore, MET, as a key molecule for tumor progression, may regulate cell biological functions through the STAT3/Akt signaling pathway. Myeloid cell leukemia-1 (MCL-1), a member of the Bcl-2 gene family, is highly expressed in many tumor tissues and participates in regulating multidrug resistance [20, 21]. A previous study showed that targeted inhibition of MCL-1 might promote DDP-induced mitochondrial apoptosis, thereby enhancing DDP sensitivity in NSCLC [22]. In addition, genipine had been suggested to promote mitochondrial dysfunction in gastric cancer by inactivating of STAT3/MCL-1 axis [23]. Therefore, we speculated that MET mediated the STAT3/Akt signaling pathway to regulate MCL-1 expression, thereby participating in the DDP resistance of NSCLC.

This study aimed to reveal the underlying molecular mechanism by which anlotinib inhibited chemoresistance in NSCLC and to provide new ideas for NSCLC treatment. Combined with the above, we proposed the hypothesis that anlotinib might repress DDP resistance in NSCLC through the MET/STAT3/Akt/MCL-1 pathway.

## 2. Methods

### 2.1. Cell Culture and Treatment

A549 and A549/DDP cells were purchased from Procell (Wuhan, China) and cultured at 37°C with 5% CO_2_ in Ham's F-12K (PM150910, Procell) containing 10% FBS (164210-50, Procell) and 1% Penicillin-Streptomycin Solution (PB180120, Procell). Besides, H1299 and H1299/DDP (Biovector, Beijing, China) were cultured in RPMI-1640 medium plus 10% FBS and 1% Penicillin-Streptomycin Solution. In functional experiments, A549/DDP and H1299/DDP cells were treated with 1 *μ*M anlotinib (CTTQ-PHARMA, Jiangsu, China), 2 *μ*M DDP (Sigma-Aldrich, St. Louis, MO, USA), or 1 *μ*M anlotinib+2 *μ*M DDP for 24 h.

### 2.2. Cell Transfection

MET overexpression vector (oe-MET), the lentivirus short hairpin RNA (shRNA) against MET or MCL-1 (shMET or shMCL-1), and their controls (oe-NC and shNC) were synthesized from Ribobio (Guangzhou, China). They were transfected into cells with lipofectamine 3000 (Invitrogen, Carlsbad, CA, USA). 24 h later, cells were treated with 1 *μ*M anlotinib, 2 *μ*M DDP, or 20 *μ*M ACT001 (Accendatech Co., Ltd., Tianjin, China) for 24 h.

### 2.3. Cell Counting Kit 8 (CCK8) Assay

A549/DDP and H1299/DDP cells were cultured in 96-well plates and then treated with anlotinib or DDP for 24 h at the indicated concentrations. After that, cells were incubated with CCK8 solution (Dojindo, Kumamoto, Japan), and then cell viability was assessed at 450 nm by using a microplate reader. In functional experiments, transfected and treated A549/DDP and H1299/DDP cells were seeded into 96-well plates and then hatched with CCK8 solution to measure cell viability.

### 2.4. Colony Formation Assay

A549/DDP and H1299/DDP cells were plated in 6-well plates and cultured for 14 days. Afterwards, cells were fixed with 4% paraformaldehyde (Beyotime) and stained with crystal violet (Beyotime, Shanghai, China). The number of colonies was counted under a microscope to assess cell proliferation.

### 2.5. Transwell Assay

A549/DDP and H1299/DDP cells suspended with a serum-free medium were seeded into the top of transwell chambers coated with or without Matrigel (for detecting cell invasion or migration, respectively). Then, a complete medium was added to the lower chamber. After 24 h, the migrated and invaded cells were fixed with 4% paraformaldehyde and stained with crystal violet, followed by counted under a microscope.

### 2.6. Quantitative Real-Time PCR (qRT-PCR)

Total RNAs were extracted with the help of the TRIzol reagent (Invitrogen), and cDNA was obtained using cDNA Synthesis Kit (Takara, Dalian, China). Then, qRT-PCR was performed with SYBR Green (Takara). Relative expression was analyzed by the 2^−ΔΔCt^ method with GAPDH as internal control. The primer sequences are listed as follows: MET, F 5′-GTCCCCAATGACCTGCTG-AA-3′, R 5′-ACATCATGAGAGGAATGCAGGA-3′, MCL-1, F 5′-TGCTTCGGAAACTGGACATCA-3′, R 5′-TAGCCA-CAAAGGCACCAAAAG-3′, GAPDH, F 5′-CTCTGCTCC-TCCTGTTCGAC-3′, and R 5′-CGACCAAATCCGTTGACT-CC-3′.

### 2.7. Western Blot Analysis

RIPA buffer (Beyotime) was utilized to extract total proteins. Protein samples were separated by 10% SDS-PAGE gel and transferred to PVDF membranes (Beyotime). After blockage with nonfat milk or BSA (CST, Danvers, MA, USA), membranes were hatched with anti-MET (#450, 1 : 1000, CST), anti-p-MET (#3126, 1 : 1000, CST), anti-STAT3 (#12640, 1 : 1000, CST), anti-p-STAT3 (#9145, 1 :2000, CST), anti-Akt (#9272, 1 : 1000, CST), anti-p-Akt (#4060, 1 : 2000, CST), or anti-GAPDH (#3683, 1 : 1000, CST) followed by treatment with the anti-rabbit IgG antibody (#7074, 1 : 3000, CST). A protein signal was detected by using SignalFire™ ECL reagent (#6883, CST).

### 2.8. Statistical Analysis

Data are expressed as the mean ± SD. GraphPad Prism 7.0 was utilized for data analysis. Differences were calculated using Student's *t*-test or ANOVA. *P* < 0.05 was considered significant difference.

## 3. Results

### 3.1. Anlotinib Inhibits DDP Resistance in NSCLC Cells

**Figure 1:**
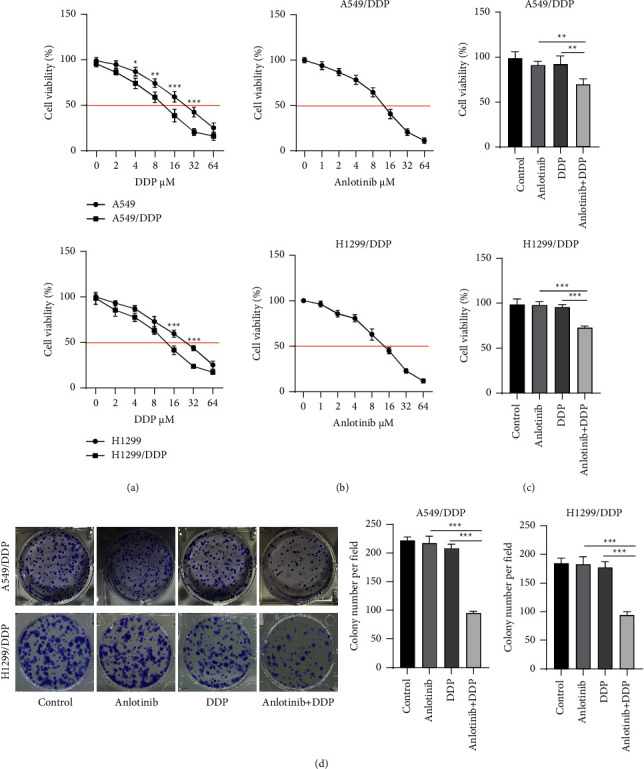
Anlotinib regulates DDP resistance in NSCLC cells. (a) The viability of A549, A549/DDP, H1299, and H1299/DDP cells under different concentrations of DDP was analyzed by using the CCK8 assay to assess the IC50 value for DDP in cells. (b) Cell viability was detected by using the CCK8 assay under different concentrations of anlotinib to assess the IC50 value for anlotinib in A549/DDP and H1299/DDP cells. (c, d) A549/DDP and H1299/DDP cells were treated with 1 *μ*M anlotinib, 2 *μ*M DDP, or 1 *μ*M anlotinib + 2 *μ*M DDP. (c) Cell viability was measured by using the CCK8 assay. (d) Colony formation assay was used to assess A549/DDP and H1299/DDP cell proliferation. ^*∗∗*^*P* < 0.01, ^*∗∗∗*^*P* < 0.001.

To confirm the DDP resistance of A549/DDP and H1299/DDP cells, we assessed cell viability under different concentrations of DDP treatment. As shown in Figure 1(a), with the increasing DDP concentration, the viability of A549 and H1299 cells was significantly lower than that of A549/DDP and H1299/DDP cells, respectively. By detecting the medium inhibitory concentration (IC50) of DDP on cells, we confirmed that A549/DDP and H1299/DDP cells had higher DDP resistance than A549 and H1299 cells. In this, 2 *μ*M DDP had no significant effect on cell viability, so it was used for subsequent functional experiments. In addition, we explored the optimal concentration of anlotinib-treated A549/DDP cells and confirmed that 1 *μ*M anlotinib had no significant effect on cell viability and could be used in subsequent functional experiments. After cells were treated with 1 *μ*M anlotinib and 2 *μ*M DDP, we determined that the viability of A549/DDP and H1299/DDP cells was significantly reduced (Figure 1(c)), indicating that anlotinib might inhibit DDP resistance of A549/DDP and H1299/DDP cells. Furthermore, we measured cell proliferation using the colony formation assay and confirmed that anlotinib and DDP alone had no significant effect on cell proliferation, while the combination of the two treatments markedly reduced cell proliferation (Figure 1(d)). Above all, anlotinib reduced DDP resistance in A549/DDP and H1299/DDP cells.

### 3.2. Anlotinib Suppresses MET Expression to Regulate the DDP Resistance of NSCLC Cells

**Figure 2:**
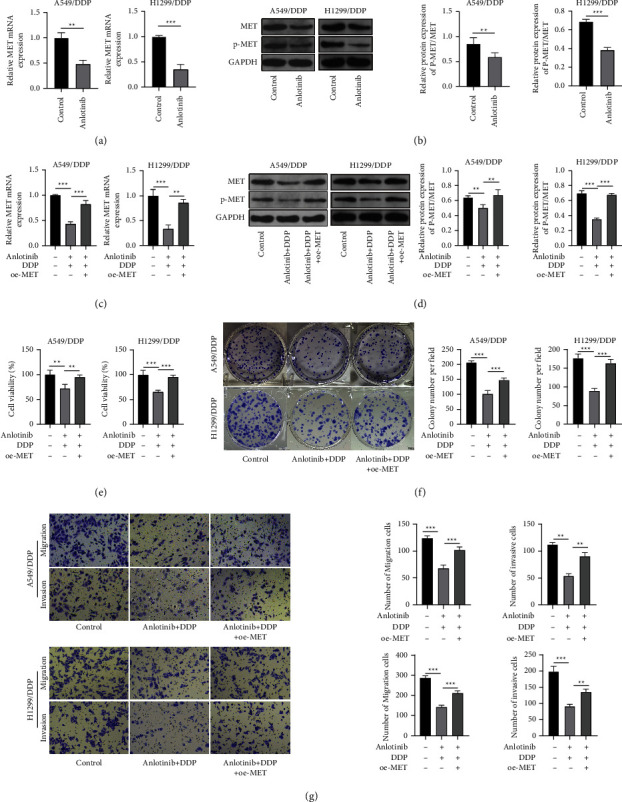
Anlotinib inhibited MET to participate in the DDP resistance of NSCLC cells. (a) MET mRNA expression was detected by qRT-PCR in A549/DDP and H1299/DDP cells treated with or without anlotinib. (b) The p-MET/MET protein expression was examined by western blot analysis in A549/DDP and H1299/DDP cells treated with or without anlotinib. (c–g) A549/DDP and H1299/DDP cells were transfected with oe-MET and then treated with anlotinib and DDP. (c) MET mRNA expression was examined by qRT-PCR. (d) Western blot analysis was used to examine p-MET/MET protein expression. CCK8 assay (e), colony formation assay (f), and transwell assay (g) were used to assess cell viability, proliferation, migration, and invasion. ^*∗*^*P* < 0.05, ^*∗∗*^*P* < 0.01, and ^*∗∗∗*^*P* < 0.001.

Anlotinib treatment significantly inhibited MET mRNA expression (Figure 2(a)). At the protein level, the expression of p-MET/MET also was reduced after anlotinib treatment (Figure 2(b)). To explore whether anlotinib restrained DDP resistance in NSCLC cells by regulating MET expression, we performed the rescue experiments. Firstly, oe-MET was transfected in A549/DDP and H1299/DDP cells to overexpress the MET level and phosphorylation. Then, A549/DDP and H1299/DDP cells were transfected with oe-MET and then treated with anlotinib and DDP. Our data showed that anlotinib and DDP treatment markedly repressed MET mRNA expression and p-MET/MET protein expression, while these effects could be abolished by oe-MET (Figures 2(c) and 2(d)). In functional experiments, anlotinib and DDP treatment significantly repressed cell viability, the number of colonies, as well as the numbers of migrated and invaded cells. However, these effects were partially eliminated by MET overexpression (Figures 2(e)–2(g)). All data showed that anlotinib inhibited MET expression and phosphorylation to suppress the DDP resistance of NSCLC cells.

### 3.3. MET Mediates the Activity of STAT3/Akt Pathway and MCL-1 Expression

**Figure 3:**
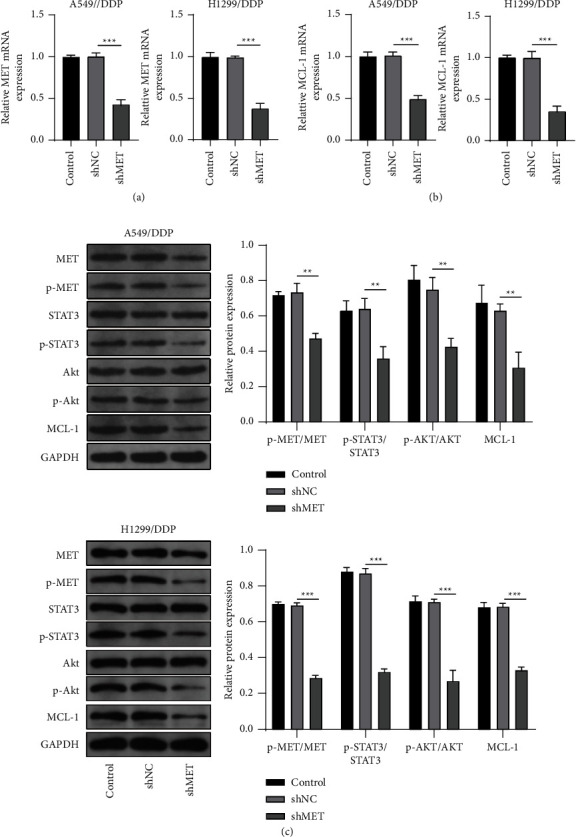
MET mediates the STAT3/Akt pathway and MCL-1 expression. A549/DDP and H1299/DDP cells were transfected with shNC or shMET. (a, b) MET and MCL-1 mRNA expression levels were examined by qRT-PCR. (c) Western blot analysis was used to test the protein expression of p-MET/MET, p-STAT3/STAT3, p-Akt/Akt, and MCL-1. ^*∗∗*^*P* < 0.01 and ^*∗∗∗*^*P* < 0.001.

Then, we investigated the downstream pathway of MET. The shMET was used to reduce MET expression in A549/DDP and H1299/DDP cells (Figure 3(a)). By detecting MCL-1 mRNA expression, we confirmed that MET knockdown remarkably decreased MCL-1 expression (Figure 3(b)). Western blot analysis results indicated that shMET significantly reduced p-MET/MET, p-STAT3/ATAT3, p-Akt/Akt, and MCL-1 protein expression (Figure 3(c)). These data revealed that MET was involved in mediating the activation of the STAT3/Akt signaling pathway to regulate MCL-1 expression.

### 3.4. MET Participates in the Regulation of Anlotinib on DDP Resistance in NSCLC Cells through Regulating MCL-1 Level

**Figure 4:**
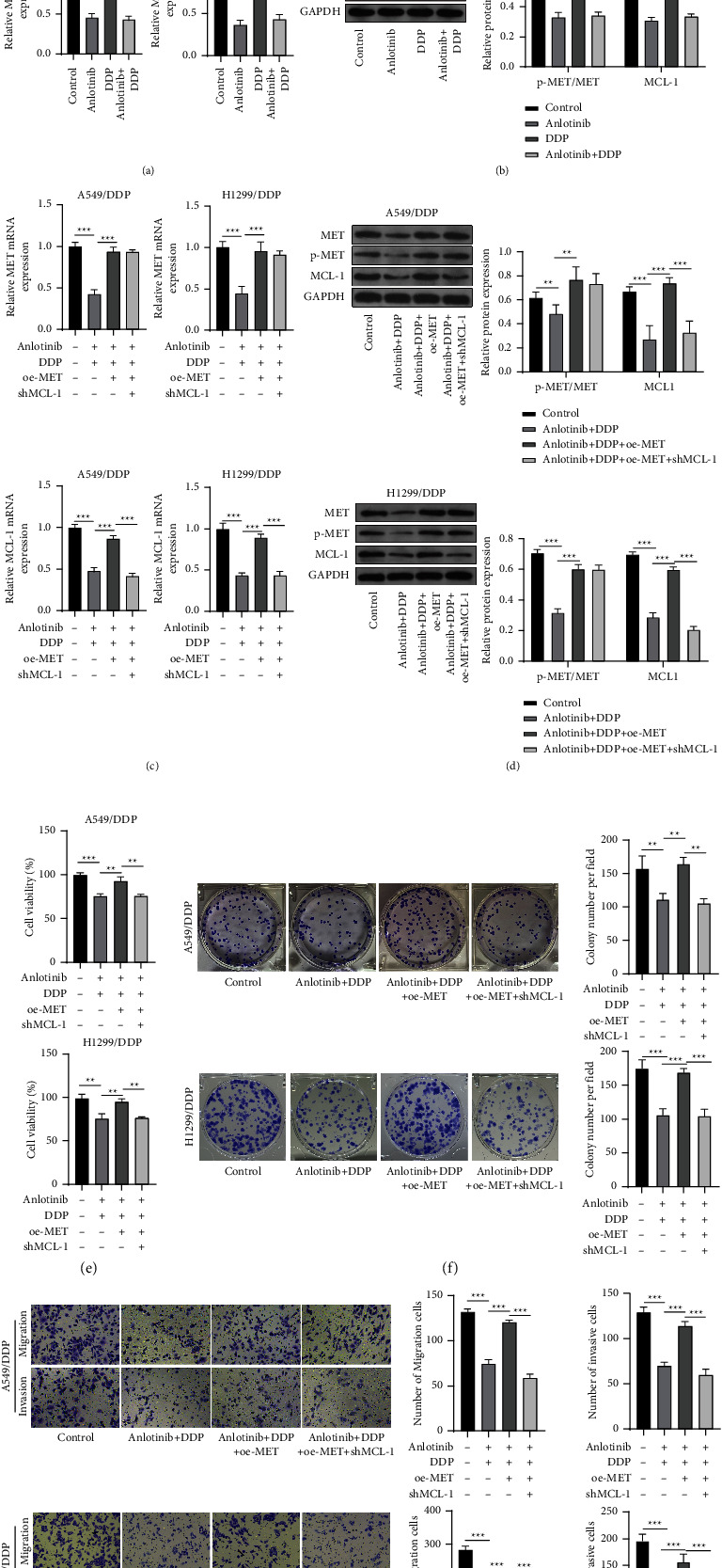
Effects of oe-MET and shMCL-1 on anlotinib-mediated DDP resistance in NSCLC cells. (a, b) A549/DDP and H1299/DDP cells were treated with 1 *μ*M anlotinib, 2 *μ*M DDP, or 1 *μ*M anlotinib+2 *μ*M DDP. (a) MET and MCL-1 mRNA expression was determined by qRT-PCR. (b) The p-MET/MET and MCL-1 protein expression was examined by western blot analysis. (c–g) A549/DDP and H1299/DDP cells were transfected with oe-MET-1 and sh-MCL-1, followed by treatment with 1 *μ*M anlotinib and 2 *μ*M DDP. (c) qRT-PCR was performed to examine MET and MCL-1 mRNA expression. (d) Western blot analysis was carried out to detect p-MET/MET and MCL-1 protein expression. Cell viability, proliferation, migration, and invasion were evaluated using the CCK8 assay (e), colony formation assay (f), and transwell assay (g). ^*∗*^*P* < 0.05, ^*∗∗*^*P* < 0.01, and ^*∗∗∗*^*P* < 0.001.

MET and MCL-1 mRNA expression, as well as p-MET/MET and MCL-1 protein expression, were significantly inhibited in both anlotinib group and anlotinib + DDP group, while DDP alone treatment had no effect on this (Figures 4(a) and 4(b)). To explore whether anlotinib regulated DDP resistance in NSCLC cells by the MET/MCL-1 pathway, we performed rescue experiments. MCL-1 mRNA and protein expression levels were reduced by shMCL-1 in A549/DDP and H1299/DDP cells. Then, A549/DDP and H1299/DDP cells were cotransfected with oe-MET and shMCL-1, followed by treatment with anlotinib and DDP. Through qRT-PCR and western blot analysis, we confirmed that oe-MET markedly enhanced MET and MCL-1 mRNA expression, as well as p-MET/MET and MCL-1 protein expression in A549/DDP and H1299/DDP cells treated with anlotinib and DDP, while the addition of shMCL-1 only suppressed MCL-1 mRNA and protein expression without affecting MET mRNA expression and p-MET/MET protein expression (Figures 4(c) and 4(d)). These data also confirmed that MCL-1 was the downstream target of MET, and its expression was regulated by MET. Moreover, we found that the promotion effects of oe-MET on cell viability, proliferation, migration, and invasion in anlotinib and DDP-induced A549/DDP and H1299/DDP cells also were reversed by MCL-1 knockdown (Figures 4(e)–4(g)). All data showed that anlotinib suppressed DDP resistance in NSCLC cells through regulating MET/MCL-1 axis.

### 3.5. Anlotinib Regulates MET/STAT3/Akt/MCL-1 Axis to Inhibit DDP Resistance in NSCLC Cells

**Figure 5:**
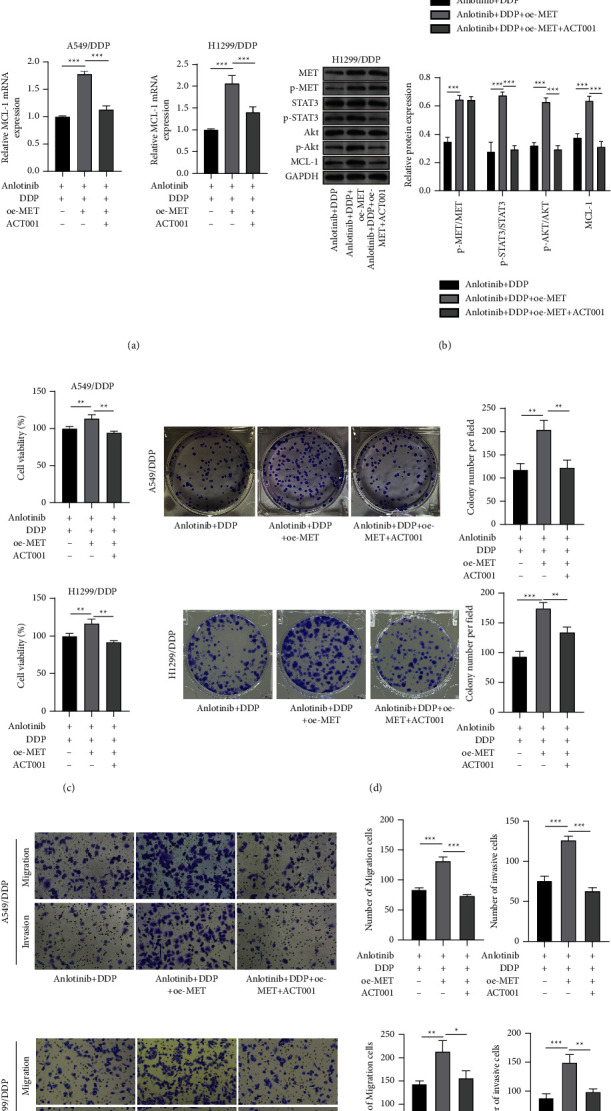
Effects of oe-MET and ACT001 on anlotinib-mediated DDP resistance in NSCLC cells. A549/DDP and H1299/DDP cells were transfected with oe-MET-1 and then treated with anlotinib, DDP, and ACT001. (a) The mRNA expression levels of MET and MCL-1 were assessed by qRT-PCR. (b) The protein levels of p-MET/MET, p-STAT3/STAT3, p-Akt/Akt, and MCL-1 were measured by western blot analysis. CCK8 assay (c), colony formation assay (d), and transwell assay (e) were performed to examine cell viability, proliferation, migration, and invasion. ^*∗∗*^*P* < 0.01 and ^*∗∗∗*^*P* < 0.001.

To explore whether anlotinib regulated MCL-1 by mediating the activity of the STAT3/Akt pathway via MET, A549/DDP and H1299/DDP cells were transfected with oe-MET and then treated with anlotinib, DDP, and ACT001. Overexpressed MET enhanced MET and MCL-1 mRNA expression, as well as p-MET/MET, p-STAT3/STAT3, p-Akt/Akt, and MCL-1 protein expression, while the addition of ACT001 only reduced p-STAT3/STAT3, p-Akt/Akt, and MCL-1 expression (Figures 5(a) and 5(b)). Function analysis showed that ACT001 treatment reversed the enhancing effect of MET overexpression on A549/DDP and H1299/DDP cell viability, proliferation, migration, and invasion under anlotinib and DDP conditions (Figures 5(c)–5(e)). These results indicated that anlotinib suppressed MET expression to mediate the activity of the STAT3/Akt pathway, thereby decreasing the MCL-1 level to inhibit DDP resistance in NSCLC cells.

## 4. Discussion

Chemotherapy is one of the main treatment methods for NSCLC, but the emergence of chemoresistance greatly reduces the efficacy of chemical drugs [5]. Therefore, it is urgent to clarify the potential mechanisms affecting chemoresistance in NSCLC and provide new ideas for overcoming NSCLC chemoresistance. Targeted therapy is characterized by strong pertinence and small side effects and has shown great advantages in cancer treatment in recent years [24, 25]. Anlotinib is an antiangiogenic targeted therapy approved for treating locally advanced, metastatic NSCLC in patients who have progressed or relapsed following at least two prior regimens of systemic chemotherapy [26, 27]. Previous study had suggested that anlotinib could overcome multidrug resistance in many cancers [16, 28]. More importantly, a recent study shows that anlotinib combined with platinum-chemotherapy has effective antitumor activity as the first-line treatment for extensive-stage small-cell lung cancer [29]. In our study, we demonstrated that anlotinib suppressed DDP resistance in NSCLC cells by regulating cell proliferation and metastasis. In terms of mechanism, we confirmed that anlotinib decreased MET expression to inactivate the STAT3/Akt pathway, thereby reducing MCL-1 expression.

MET plays a proto-oncogene role in many cancers, and activation of the MET-related pathway plays a vital role in cancer development [30, 31]. Multiple previous research studies had indicated that MET amplification might lead to EGFR-TKI resistance, and its inhibition could overcome resistance to EGFR-TKI in NSCLC [32]. Anlotinib had been confirmed to restrain cancer cell chemoresistance via blocking MET expression and phosphorylation [14, 16]. Consistent with these results [14, 16], we pointed out that anlotinib repressed DDP resistance in NSCLC via decreasing MET expression. STAT3/Akt is a key pathway regulating tumor progression and is closely related to the occurrence of many tumors, such as neuroblastoma [33] and cervical cancer [34]. It has been reported that MET is involved in regulating the cancer malignant phenotype by STAT3 and Akt [14, 18, 19]. Here, we found that MET knockdown inhibited the activity of STAT3/Akt pathway, and targeted inhibition of the STAT3/Akt pathway using ACT001 could abolish the promotion effect of MET on anlotinib-mediated DDP resistance in NSCLC cells. The above data further revealed that MET regulated the STAT3/Akt pathway to participate in the regulation of anlotinib on DDP resistance in NSCLC.

MCL-1 may be a key molecule leading to drug resistance in patients, and it is involved in regulating apoptosis and chemoresistance in cancers [20, 35]. Studies had shown that MCL-1 was highly expressed in NSCLC, and its inhibition reduced NSCLC cell proliferation and DDP resistance [36, 37]. Novel SHP2 degrader SHP2-D26 in combination with the EGFR inhibitor osimertinib (AZD9291) could overcome osimertinib resistance in NSCLC by reducing MCL-1 expression [38]. These evidences suggested that a high expression of MCL-1 was associated with drug resistance in NSCLC. In this, MCL-1 expression could be reduced by anlotinib and MET knockdown. Functional experiments showed that MCL-1 knockdown reversed the enhancing effect of MET on anlotinib-mediated DDP resistance, confirming that anlotinib might suppress DDP resistance by regulating MCL-1 through MET. Besides, we also found that STAT3/Akt pathway inhibitor ACT001 could decrease MCL-1 mRNA and protein expression, which further indicated that MET regulated the STAT3/Akt pathway to mediate MCL-1 expression.

## 5. Conclusions

Our study suggests a new mechanism by which anlotinib inhibits chemoresistance in NSCLC. Our research pointed out that anlotinib restrained DDP resistance in NSCLC through the MET/STAT3/Akt/MCL-1 pathway. The discovery of MET/STAT3/Akt/MCL-1 axis provides a new idea for overcoming DDP resistance in NSCLC and has important clinical significance.

## Data Availability

The authors can make data available on request through a data access committee and institutional review board. In addition, all the data can also be obtained from the author Lile Wang.

## Abbreviations

NSCLC: Non-small-cell lung cancer
DDP: Cisplatin
TKI: Tyrosine kinase inhibitor
MET: Mesenchymal-epithelial transition factor
MCL-1: Myeloid cell leukemia-1
CCK8: Cell counting kit 8
qRT-PCR: Quantitative real-time PCR.

## Additional Points

*Highlight*. 1. Anlotinib suppresses DDP resistance in NSCLC by decreasing MET expression. 2. MET mediates the STAT3/Akt pathway to regulate the MCL-1 level. 3. Anlotinib regulates the MET/STAT3/Akt/MCL-1 pathway in NSCLC.

## Supplementary Materials

Supplementary Materials In A549/DDP and H1299/DDP cells, we assessed the efficacy of oe-MET for high expression of MET and shMCL-1 for low expression of MCL-1. Oe-MET was transfected in A549/DDP and H1299/DDP cells to overexpress the MET level and phosphorylation. MCL-1 mRNA and protein expression levels were reduced by shMCL-1 in A549/DDP and H1299/DDP cells.
